# The Design and Optimization of an Acoustic and Ambient Sensing AIoT Platform for Agricultural Applications

**DOI:** 10.3390/s23146262

**Published:** 2023-07-10

**Authors:** Ahmed Alzuhair, Abdullah Alghaihab

**Affiliations:** Department of Electrical Engineering, College of Engineering, King Saud University, P.O. Box 800, Riyadh 11421, Saudi Arabia; aalghaihab@ksu.edu.sa

**Keywords:** IoT, machine learning, TinyML, artificial intelligence, efficient sensor nodes, low-power communication

## Abstract

The use of technology in agriculture has been gaining significant attention recently. By employing advanced tools and automation and leveraging the latest advancements in the Internet of Things (IoT) and artificial intelligence (AI), the agricultural sector is witnessing improvements in its crop yields and overall efficiency. This paper presents the design and performance analysis of a machine learning (ML) model for agricultural applications involving acoustic sensing. This model is integrated into an efficient Artificial Intelligence of Things (AIoT) platform tailored for agriculture. The model is then used in the design of a communication network architecture and for determining the distribution of the computing load between edge devices and the cloud. The study focuses on the design, analysis, and optimization of AI deployment for reliable classification models in agricultural applications. Both the architectural level and hardware implementation are taken into consideration when designing the radio module and computing unit. Additionally, the study encompasses the design and performance analysis of the hardware used to implement the sensor node specifically developed for sound classification in agricultural applications. The novelty of this work lies in the optimization of the integrated sensor node, which combines the proposed ML model and wireless network, resulting in an agricultural-specific AIoT platform. This co-design enables significant improvements in the performance and efficiency for acoustic and ambient sensing applications.

## 1. Introduction

Agriculture has undergone significant transformations over the last century compared to early primitive farming with very limited capabilities. The use of technology such as improved tools, machinery, and automation has led to significant improvements in agriculture, resulting in increased yields, quality, and efficiency. Most recently, agriculture has been undergoing another major transition through the increased utilization of internet of things (IoT) devices and artificial intelligence (AI), which, together, have the potential to make a substantial impact on the agricultural sector [1]. According to a recent report [2], the use of the IoT in agriculture is expected to grow from $12.5 billion in 2021 to $28.6 billion in 2030, whereas the AI agriculture market is expected to reach $2.5 billion in 2026, from $0.77 billion in 2020, with an impressive compound annual growth rate of 21.5% [3]. If the trend continues, these combined markets are expected to reach $34.1 billion in 2030. Agriculture benefits from IoT devices’ ability to sense, process, and transmit environmental data, such as vision, acoustic, and ambient data, including temperature, soil humidity, and nutrition, from usually vast distributed lands. AI, on the other hand, can be trained to analyze sensor data, improving crop and livestock wellness and management by making informed decisions about irrigation, fertilization, and pest and animal control [4,5]. Cost is a major factor limiting AIoT market adoption in general and particularly for non-corporate farmers. This can be improved by smarter AI algorithms relying only on less expensive acoustic and ambient sensors powered by inexpensive hardware. Vision sensors require more complex hardware to process, are prone to damage, and need more cleaning compared to acoustic and ambient sensors to maintain image quality. Furthermore, not only are operational expenses increased due to frequent maintenance, but major capital costs are expected since cameras are more expensive and require proper installation and mounting. Therefore, they are considered to be less suitable for agricultural applications with massively distributed AIoT devices.

Common to most IoT applications, data are aggregated from many devices, which results in platform architecture challenges. One approach is to transmit these data continuously to the on-premises or cloud server for subsequent processing. Alternatively, complete or partial processing is performed at the edge utilizing the IoT device’s on-board computational capabilities, limiting transmission to critical events. The computational load allocation between the edge and server must be driven by the application’s realistic use cases, such as data, AI model complexity, and event frequency, in addition to the hardware cost and efficiency. In this work, we design and discuss realistic proof-of-concept AI models for acoustic and ambient sensing agricultural applications, which are utilized to inform the network architecture design and computational load allocation between the edge devices and cloud for an efficient AIoT platform.

## 2. Background and Related Work

At the architectural level, the IoT can be divided into IoT devices, the communication network, and a central server hosting the application layer [6]. Unlike the traditional IoT, AIoT or edge devices and servers can perform AI computations. To power AI, edge devices may contain powerful processing, such as dedicated graphical processing units (GPUs), or could simply be powered by very cost- and energy-efficient microprocessor units (MCU). These devices come in various form factors and can be hosted on advanced unmanned aerial vehicles [7] or ground stationary or in-motion ground units, depending on the applications’ requirements and economics. The backbone of the network may include custom communication links to support data aggregation to the central server, which may be hosted locally or in cloud services, depending on privacy and cost considerations. The application layer, at the top of the architectural paradigm, manages the platform’s services. For example, in agricultural applications, it could provide farmers with analytics derived from the distributed environmental sensors and manual and automatic control mechanisms.

Besides the traditional ambient sensors, there has been an increased utilization of sound and vision technologies in agricultural sensing. Acoustic sensors have been used for monitoring plant health by detecting and estimating pest populations [8,9,10], and in agricultural machinery health monitoring [11]. Most recently, it was shown that stressed plants emit ultrasonic airborne sounds that can be utilized by machine learning algorithms to detect their hydration level or injury [12]. Vision sensors have also been widely employed to monitor plant health and detect diseases [13]. Furthermore, it has been shown that deep learning is quite effective in detecting plant leaf disease [14,15]. However, increased sensor capabilities generally come at the expense of increased platform costs and complexity.

In [16], a hierarchical federated learning approach based on semi-synchronous communications was introduced for heterogeneous IoT edge environments. Wi-Fi was used as the communication network, which would limit its range and restrict its use in applications where long-range communication is needed. Deep neural networks were used in [17] to acoustically monitor bee activities. Both edge and in-cloud computing architectures were considered without an in-depth analysis. A cellular network was proposed to connect the sensor nodes to the cloud; however, it would require rapid battery charging or replacements to run the wireless communication module.

Moreover, ref. [18] presented an urban noise-monitoring system using deep learning implemented on the edge using a Raspberry pi 4. However, the paper did not discuss the network architecture. An outline for an IoT device architecture to be used in cellular biology was presented in [19]. The needed infrastructure for communicating, storing, and processing the datasets was analyzed at an abstract level. 

The work presented in [16,17,18,19] lack an analysis of the impact of the hardware design on the overall system performance, especially with respect to the average power consumption. In [20], a low power network was proposed for smart agriculture based on the IEEE 802.15.4 standard. The paper proposed using a LoRa-based gateway to connect it to the cloud. In addition, various off-the-shelf processing and radio units were compared in terms of their costs and power consumption. However, AI deployment was not demonstrated or analyzed to complete the design in order to achieve the goals of smart agriculture. Similarly, ref. [21] demonstrated a mesh-networking protocol for real-time air quality monitoring without incorporating AI to improve its performance. 

In this paper, we design, analyze, and optimize the AI deployment of sound classification models in agriculture applications. The design includes both the architectural level and hardware implementation consideration, which is applied to the computational unit and radio module. A machine learning (ML)-based model for sound classification in agricultural applications is designed and its performance is analyzed assuming a certain selected hardware implementation. The model is designed to classify the following classes: rain, fire, sheep, and insects, which are selected to encompass the diverse agricultural environment. The targeted applications include rain and flood monitoring, crop fire prevention, livestock monitoring and management, and plant health monitoring.

The organization of this work is as follows: Section 3 presents the platform design including the architecture, network, machine learning model, and edge device designs. Section 4 presents and discusses the results. Section 5 concludes this work.

## 3. Platform Design

### 3.1. Architecture

**Figure 1:**
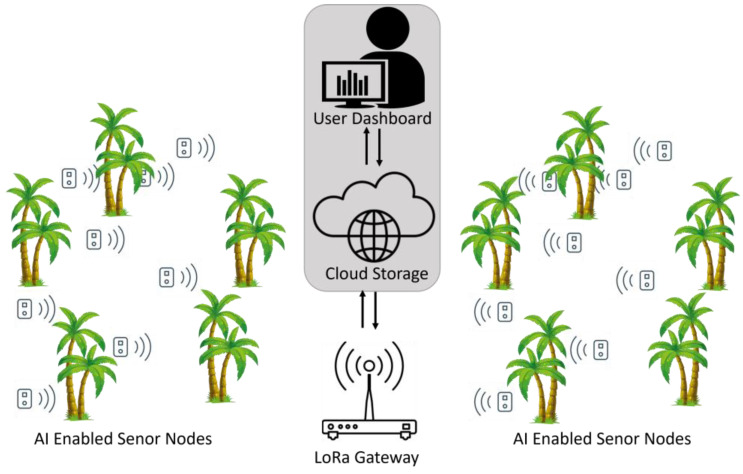
System architecture.

Figure 1 shows the simplified platform architecture used for the sound classification. The platform consists of distributed sensor nodes connected through a LoRa network to the main gateway. Each sensor node includes mainly acoustic and ambient sensors, an RF module, a processing unit, and a battery. The gateway is linked to the internet to allow for cloud data storage and analytics. This can be displayed to the end-user using a user-friendly graphical interface, which helps to efficiently monitor the area and make informed decisions. The system can be scaled up with minimal additional cost, as it is designed to support a high number of sensor nodes simultaneously. 

### 3.2. Communication Network Design

The power consumption of the RF wireless communication module represents a significant portion of the power consumed in wireless sensor nodes. As a result, optimizing the communication network is an integral component in the design of energy-efficient wireless sensor nodes for the IoT in agriculture. The communications should not only meet the energy-stringent requirements of the system, but also the data rates and communication range specifications. 

Considering the IoT sensor nodes’ applications in agriculture, the communication network should be designed to support a long-range link to cover the wide area nature of most industry-scale farms. In addition, the network deployment cost should be low enough to improve the overall economics for the farmers and their agriculture business. This can be supported by using an existing standard compliant communication protocol to take advantage of the existing infrastructure and resources, facilitating compatibility with commercially available products. Moreover, the network needs to be secure in order to protect the operation of the farms and their sensitive data. LoRa (short for Long Range) is a low-power, long-range wireless communication technology that has gained a lot of attention in terms of its deployment in IoT (Internet of Things) applications. In addition to its energy efficiency, it is a standard compliant network that is low-cost, secure, and scalable [22]. 

The LoRa network is designed to support long-range communication to cover several kilometers [22]. This is achieved by designing receivers with improved sensitivity, which allows it to detect weak signals that are attenuated by distance and/or obstacles. Its extended range makes LoRa more suitable for agricultural applications where the sensor nodes are spread out over a large area and allows it to support a high number of sensor nodes simultaneously. 

One commonly used communication protocol in IoT applications is the IEEE 802.11 family of standards (Wi-Fi). However, LoRa provides a more optimized performance compared to Wi-Fi in agricultural applications for several reasons. First, Wi-Fi is designed to cover a limited area of several tens of meters only [22]. Second, Wi-Fi RF modules consume significantly more power than LoRa modules [23]. As a result, this limits their battery lifetime and increases the operational cost of the senor nodes. Third, since Wi-Fi shares its frequency band with other standards, such as Bluetooth and Zigbee, it is more susceptible to interference. Finally, although Wi-Fi supports much higher data rates compared to LoRa, this is not needed for sensor nodes used in agricultural applications. In particular, these senor nodes only need to transmit small packets of data over long distances, especially with edge computing, as will be explained later in this paper. 

Sigfox is another alternative low-power, wide-area network (LPWAN) standard similar to LoRa. However, unlike LoRa, it is based on a proprietary network provided by Sigfox [24]. This limits the flexibility in the network design and increases the overall cost of deploying the sensor node devices. Consequently, selecting LoRa is a lower-cost and more flexible option for developing an open architecture communication network. Table 1 summarizes the performances of LoRA, Wi-Fi, Sigfox, and NB-IoT. The selected network topology is a star network. This helps in reducing the deployment cost and overhead and allows for a high scalability of the network. 

### 3.3. ML Model Design

The publicly available environmental sound dataset in [25] was employed for the model training, validation, and testing. It consists of 50 classes with 40 labeled recordings (5 s long each) per class. The following classes: rain, fire, sheep, and insects, were selected to represent the agricultural environment. To simulate real-time acquisition in their practical deployment, a 1.5 s running window with a 53% overlap was applied to the recordings and the output of the simulated buffer was next fed to the model. Therefore, the resulting number of cropped recordings or sample size was 240 samples per class. The samples were divided into 60% training, 20% validation, and 20% testing.

**Figure 2:**
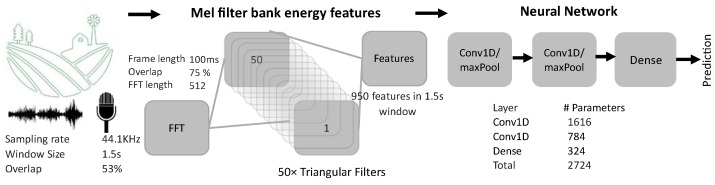
Machine learning and sound pre-processing pipeline.

Various pre-processing algorithms and neural network architectures were explored to arrive at the selected ML model for sound classification in agricultural applications. The designed machine learning pipeline consisted of a preprocessing step for the feature extraction, which was followed by a neural network for the classification, as depicted in Figure 2. Mel-filter bank features were selected for the preprocessing, which decomposed the signal into Mel-scale non-linear frequency bands. The output of the running window, 1.5 s per sample, was further divided into 100 ms frames with a 75% overlap to extract the Mel-filter features. The 950 Mel-filter features, per 1.5 s long sample extracted in the pre-processing step, were first reshaped into 50 columns. Next, these were fed to a cascade of a one-dimensional convolutional neural network, with a ReLU activation function and a max pooling layer (2x CNN1D/pool). Finally, the cascade was followed by a dense layer to arrive at the final sample prediction.

### 3.4. Edge Device Design

ML model deployment at the edge is challenging for resource-constrained devices such as microcontroller units (MCU). The small compute and memory resources limit the complexity of ML models. Energy consumption is also an important consideration, since devices are typically powered from a limited battery or harvested energy sources. Latency can be a hard requirement for some agricultural applications, such as crop fire prevention, and it also indirectly affects energy consumption through its ability to duty cycle to enter orders of magnitude, lower power sleep, or stop modes. This necessitates selecting efficient architectures and performing optimization techniques such as model pruning and quantization. Besides hardware challenges, edge devices typically have limited software development kits (SDK) without ML optimization and deployment support, which significantly slows the machine learning operation (MLOps) workflow.

Edge Impulse [26,27] is an MLOps tool for designing and optimizing machine learning algorithms for resource-constrained hardware, most recently known as TinyML. The platform allows for hardware resource utilization and performance characterization, facilitating rapid-design space exploration. It also reports the RAM and FLASH memory size requirements, along with latency estimates for the selected hardware architecture. Moreover, the platform can be further used to generate the binary for the MCU programing. 

We utilized this tool to explore various architectures from the ARM cortex m family, which is suitable for the IoT and processing at edge applications. In our selection, the goal was to minimize the MCU pre-processing and neural network inference power while finishing early (to the duty cycle) or, in the worst case, in time to meet the application latency requirement. 

To estimate the consumed power, we used STMicroelectronics ARM MCU cortex m low power family datasheets and CubeMx [28]. The peripherals that our application required, such as the analog to digital converter (ADC), general-purpose inputs and outputs (GPIOs), and serial peripheral communication interface (SPI) power consumption, were added to the expected CPU core estimates. Furthermore, full RAM retention was assumed in the stop mode power estimates. Since our pipeline required 1.5 s at 44.1 kHz of buffering, sufficient storage was added to the memory estimates.

## 4. Results and Discussion

### 4.1. ML Model Edge Device Performance

**Figure 3:**
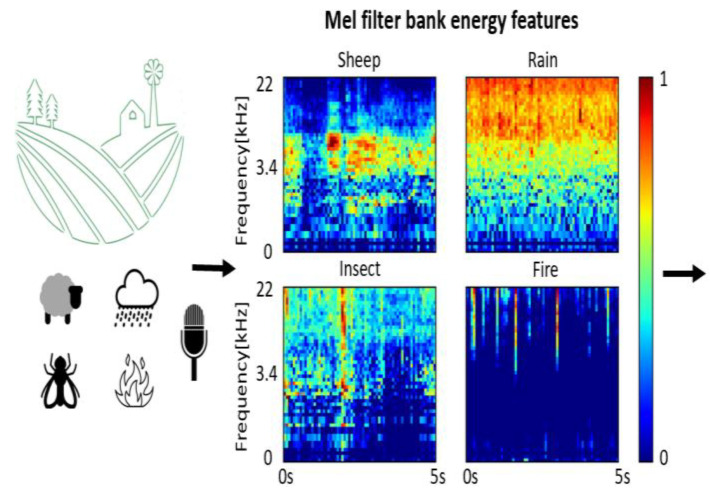
Sample output of the spectrogram for the four classes with frequency and time domain distinguishable signatures.

The designed ML model performed sound classifications in agricultural applications and served as a proof-of-concept of the environment. A sample output of the pre-processing spectrogram is shown in Figure 3 for the four classes with frequency and time domain distinguishable features, which were facilitated by the proper filter and FFT parameters selection. The achieved validation and testing performance of the consequent ML module are summarized in Table 2 and Table 3, respectively. We experimented with various neural network architectures and parameters (such as the number, dimensions, and configuration of the CNN and fully connected layers) to arrive at the selected design. Moreover, it was also observed that both Fast Fourier Transform (FFT) and raw data input features to the neural network achieved lower performances compared to the Mel filter bank features. It should be noted that some of the used sound clips included non-class associated noise or silent portions. We believe a better performance is achievable if these data are cured further, which we omitted to do in this study.

The achieved hardware performance, computation, and memory resource utilization are shown in Table 4. Compared to ARM cortex-m0 and cortex-m4, Cortex-m33 achieved the lowest average power consumption at 3.8 mW, which was facilitated by the model optimization. The reduced latency allowed for duty cycling and entering the very efficient MCU stop mode with full RAM retention, substantially increasing the energy savings. The MCU consumed only 12 µW in the stop mode with a negligible startup energy smaller than 50 nJ [28]. Although the pre-processing module added considerable computational resources, the selection was justified with the substantial performance improvement compared to the CNN-only pipeline. The 8-bit quantized model resulted in 26% power saving compared to the 32-bit float implementation, without sacrificing accuracy.

In our class choices, we attempted to include a variety of class types to represent the agricultural application. Although they were obviously not inclusive of all possible agricultural applications, we believe that our results established a reasonable baseline for the expected ML model and hardware performance, and resource utilization for agricultural applications. Furthermore, since ambient sensors such as temperature and humidity sensors are orders of magnitude slower than the acoustic sensor sampling rate, they are expected to have a negligible performance impact.

### 4.2. Overall Energy Consumption

In order to optimize the total average power consumption of the sensor nodes, both the RF and computation modules were analyzed. The analysis was based on finding each component’s contribution to the average power consumption, including the duty-cycling impact if applicable. Using in-node processing of the data acquired by the sensors, the power-hungry RF module could be turned off when the sensors’ data features did not show any possible desired characteristics. This would result in significant energy savings by reducing the average power consumption, which is calculated using the following equation [29]:

(1) $$P_{avg}=\alpha \times P_{RF_{Active}}+\left(1-\alpha \right)\times P_{RF_{Sleep}}+\beta \times P_{Compute_{Active}}+\left(1-\beta \right)\times P_{Compute_{Sleep}\;\;\;\;\;\;\;}$$

where *α* and *β* represent the on-time ratios for the RF and computation modules, respectively. The active and sleep power of each component were based on standard off-the-shelf modules. The optimization process aimed to balance the energy consumption in edge computing and during data transmission to the cloud. Since the active power of the RF wireless module was high, edge processing was used to reduce the on-time of the power-hungry RF transmitters. This optimization process is application specific; for instance, other applications require more resource-intensive computing tasks, which makes offloading these tasks by transmitting them to the gateway more efficient. The rest of our analysis in this section will be based on the specific application presented in this work, which utilized a practical ML model for acoustic and ambient sensing agricultural applications.

**Figure 4:**
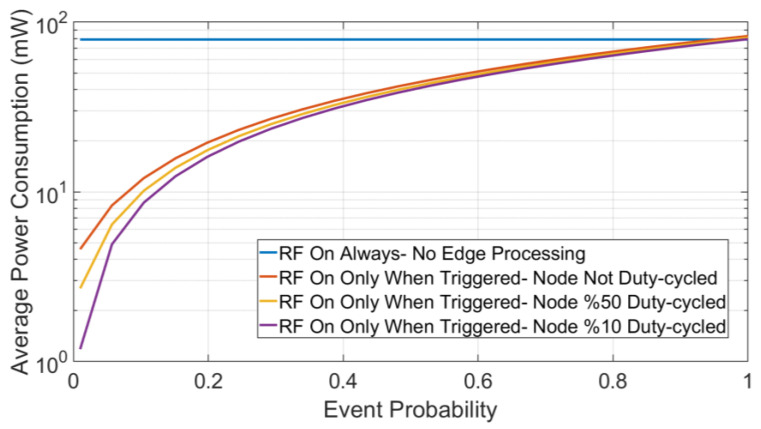
Event probability vs. average power consumption.

Figure 4 shows the resultant average power for various event frequencies, where an event is defined as when possible desired data characteristics are detected by the sensor node. In the first case, the RF module was in sleep mode by default and was only turned on when an event was detected (0 ≤ *α* ≤ 1) while the computation module was running %10 of the time. The result shows that, when the event probability was below %95, the average power consumption was below the power consumed by the RF module only with no edge processing. In the latter case, the power-hungry RF circuitry would have to stay on all the time and the data processing was performed in a central unit or the cloud. Consequently, utilizing edge computing would significantly help to increase the energy efficiency of the sensor nodes and increase the battery lifetime, especially for infrequent events. In addition, turning off the RF transmitter when not needed would help in reducing the interference in the wireless band and enhance the reliability of all the communication links in the sensor node network. 

To lower the energy consumption and increase the battery lifetime further, duty cycling the sensor node was considered. As Figure 4 demonstrates, for a low probability of event occurrence below %10, the average power consumption would be lower by at least 17% and 35% when the MCU was 50% and 10% duty cycled, respectively. Consequently, the impact of the senor node duty cycling became more visible for infrequent events.

### 4.3. Economic Impact and Scaling

AIoT platforms are expected to improve the quality of decisions concerning farm management and subsequently economics for farmers. The capital costs associated with platform implementation are considered to be small with very cost-effective and scalable MCUs and the LoRa communication architecture. The operational costs are also non-significant, as MCUs are generally reliable and the selected sensors are easy to maintain. Furthermore, ML model updates are facilitated by swappable flash memory or over-the-air (OTA) update arrangements, utilizing the existing communication architecture.

## 5. Conclusions

In this paper, we designed and evaluated a practical ML model for acoustic and ambient sensing in agricultural applications This study presented a new design that focused on enhancing the integrated sensor node by combining the suggested ML model and a wireless network to develop an optimized AIoT platform specifically tailored for agricultural applications. The ML model was used to optimize the design of the network architecture and the distribution of the computing load between edge devices and the cloud for an effective AIoT platform in agricultural applications. Overall, the design, analysis, and optimization of the critical components in a sensor node used for the AI deployment of reliable classification models for agricultural applications were covered in this study. Specifically, this work considered both the architectural level and hardware implementation when it came to the radio module and computing unit. Design and performance analyses of a machine learning (ML)-based model for sound classification in agricultural applications were presented using a specific hardware implementation selected to optimize the overall system performance. The results show that, when the desired event, which triggered turning on the RF module based on the sensed data, had a probability of occurrence of less than 95% of the time, utilizing edge intelligence was much more energy and spectrum efficient. This was because of the energy-hungry RF modules, which have a power consumption floor level even at lower data rates suitable for acoustic and ambient sensing, which LoRa, for example, can offer. The result would be substantially more evident when higher data rates are required, necessitating using RF modeling with a transmitting power consumption exceeding 1 W. On the other hand, processing the data at the edge could introduce some limitations, which vary in terms of their impact and depend on the application. For example, the edge has limited computing recourses when compared to the centralized infrastructure. Nevertheless, utilizing edge intelligence for lightweight applications, such as acoustic and ambient sensing in agricultural applications, would not impact the system performance. For other, more compute-intensive applications, other architectures such as edge–cloud collaboration can be utilized to optimize a system’s energy consumption and performance.

## Figures and Tables

**Table 1:** Comparison table of LoRA, Wi-Fi, Sigfox, and NB-IoT.

Parameter	LoRA	Wi-Fi	Sigfox	NB-IoT
**Range**	Long	Short	Long	Intermediate
**Power consumption**	Low	High	Low	High
**Interference**	Low	High	Low	Low
**Subscription cost**	No	No	Yes	Yes
**Data rate**	Low	High	Very Low	Low

**Table 2:** Validation accuracy: 95.8% Loss = 0.12.

	FIRE	INSECT	RAIN	SHEEP
**FIRE**	97.5%	2.5%	0%	0%
**INSECT**	4%	92%	4%	0%
**RAIN**	0%	2.1%	97.9%	0%
**SHEEP**	0%	3.6%	0%	96.4%
**F1**	0.96	0.92	0.97	0.98

**Table 3:** Testing accuracy: 87.5%, confidence threshold = 0.6.

	FIRE	INSECT	RAIN	SHEEP	UNCERTAIN
**FIRE**	89.6%	10.4%	0%	0%	0%
**INSECT**	16.7%	70.8%	10.4%	0%	2.1%
**RAIN**	0%	2.1%	97.9%	0%	0%
**SHEEP**	2.1%	2.1%	4.2%	91.7%	0%
**F1**	0.86	0.76	0.92	0.96	0%

**Table 4:** Hardware latency and power performance estimates.

MCU	Cortex-M33 @160 MHz	Cortex-M33 @160 MHz
**Quantized model**	Yes (8-bit)	No (float32)
Peak RAM *	99.0 KB	105.6 KB
Used clash *	52.4 KB	59 KB
Latency (processing + inference *	76 + 3 ms	76 + 35 ms
**Peripherals**	ADC, GPIO, SPI	ADC, GPIO, SPI
**VDD**	2.4 V	2.4 V
Active power **	37.5 mW	37.5 mW
Stop 3 power with RAM retention **	12.0 µW	12.0 µW
**Duty cycle**	10%	13.7%
Average consumption **	3.8 mW	5.1 mW
**Accuracy**	87.5%	87.5%

* Edge impulse estimates [26,27]. ** STM32U575CG Estimates [28].

## Data Availability

Not applicable.
